# Dataset for TiN Thin Films Prepared by Plasma-Enhanced Atomic Layer Deposition Using Tetrakis(dimethylamino)titanium (TDMAT) and Titanium Tetrachloride (TiCl_4_) Precursor

**DOI:** 10.1016/j.dib.2020.105777

**Published:** 2020-05-28

**Authors:** Woo-Jae Lee, Eun-Young Yun, Han-Bo-Ram Lee, Suck Won Hong, Se-Hun Kwon

**Affiliations:** a School of Materials Science and Engineering, Pusan National University, 30 Jangjeon-Dong Geumjeong-Gu, Busan, 609-735, Republic of Korea; b Department of Materials Science and Engineering, Incheon National University, 119 Academy-ro, Yeonsu-gu, Incheon, 22012, Republic of Korea; c Department of Cogno-Mechatronics Engineering, Department of Optics and Mechatronics Engineering, Pusan National University, 30 Jangjeon-Dong Geumjeong-Gu, Busan, 609-735, Republic of Korea

**Keywords:** Plasma-Enhanced Atomic Layer Deposition (PEALD), TiN (Titanium Nitride), tetrakis(dimethylamino)titanium (TDMAT), titanium tetrachloride (TiCl_4_)

## Abstract

A dataset in this report is regarding an article “Ultrathin Effective TiN Protective Films Prepared by Plasma-Enhanced Atomic Layer Deposition for High Performance Metallic Bipolar Plates of Polymer Electrolyte Membrane Fuel Cells” [1]. TiN (Titanium Nitride) thin films were deposited by Plasma-Enhanced Atomic Layer Deposition (PEALD) method using well known two types of precursor: using tetrakis(dimethylamino)titanium (TDMAT) and titanium tetrachloride (TiCl_4_), and plasma. Summarized reports, growth characteristics (growth rate as a function of each precursor pulse time, plasma power, precursor and plasma purge time, thickness depending on the number of PEALD cycles), each precursor structural information and the atomic force micrographs (AFM) data are herein demonstrated. For TDMAT-TiN, N_2_ plasma was used as a reactant whereas, H_2_+N_2_ plasma was used as TiCl_4_-TiN reactant. To apply the bipolar plate substrate, two types of TiN thin films were introduced into Stainless steel (SUS) 316L.

Specifications table

**Table:** 

**Subject**	Materials Chemistry, Material Science
**Specific subject area**	TiN thin films
**Type of data**	Image (atomic force microscopy-AFM), Graph (growth characteristics), Table (Previous report summary, precursor structural information)
**How data were acquired**	The film thickness was analyzed by field-emission scanning electron microscopy (FESEM, Hitachi, S-4800. Film morphology on SUS 316L was investigated by MFP-3D AFM (Asylum Research). Precursor structural information was simulated by Mavin program.
**Data format**	Raw Analyzed
**Parameters for data collection**	TiN thin films using TDMAT and TiCl_4_ are deposited at the temperature of 200 and 350 °C, respectively. Operation pressure is 3 torr.
**Description of data collection**	Growth rate was obtained by thickness (obtained by FESEM) divided by the repeated cycles
**Data source location**	School of Materials Science and Engineering, Pusan National University, 30 Jangjeon-Dong Geumjeong-Gu, Busan 609-735, Republic of Korea
**Data accessibility**	With the article
**Related research article**	Woo-Jae Lee, Eun-Young Yun, Han-Bo-Ram Lee, Suck Won Hong, and Se-Hun Kwon “Ultrathin Effective TiN Protective Films Prepared by Plasma-Enhanced Atomic Layer Deposition for High Performance Metallic Bipolar Plates of Polymer Electrolyte Membrane Fuel Cells” https://doi.org/10.1016/j.apsusc.2020.146215

## Value of the data

- The data is useful to understand the study conducted in “Ultrathin Effective TiN Protective Films Prepared by Plasma-Enhanced Atomic Layer Deposition for High Performance Metallic Bipolar Plates of Polymer Electrolyte Membrane Fuel Cells”
- This data is helpful to researcher to select the Ti precursor according to the application purpose.
- This data gives some information of deposition condition when researcher uses two kinds of the Ti precursors for ALD.

## 1 Data Description

As shown in Table 1, thermal decomposition of TDMAT precursor is occurred over 200 °C and TDMAT-TiN was deposited at less than or equal to 200 °C. On the other hands, TiCl_4_-TiN thin films have been studied over 300 °C because TiCl_4_ precursor is needed for high temperature mostly due to the problem for the incorporation of chlorine.

**Table 1:** Previous reports with regards to various properties for TiN thin films by ALD using TDMAT and TiCl_4_ precursor.

TDMAT-Thin Film	Reactant	Deposition Temperature (°C)	Density (g/cm^3^)	Impurity (at. %)				Thermal Decomposition Temperature (°C)	Resistivity (μΩcm)	Reference
				C	H	O				
TiN	H_2_ plasma	150	4.1	11	-	0		-	275∼210	J. Electrochem. Soc. 155 8 (2008) H625
TiN	NH_3_ plasma	200		<1		5		205	4,823	
∼180	Microelectron Eng. 86 (2009) 72									
TiN	NH_3_ plasma	150	3.5	-	-	-		-	-	ACS Appl. Mater. Interfaces 6 (2014) 7316
TiN	NH_3_	170-190	3.55	25				200	<1,000	Jpn. J. Appl. Phys. 42 (2003) 4245
TiCl_4_-Thin Film	Reactant	Deposition Temperature (°C)	Density (g/cm^3^)	Impurity (at. %)				Thermal Decomposition Temperature (°C)	Resistivity (μΩcm)	Reference
				C	H	O	Cl			
TiN	N_2_, H_2_ plasma	100	-	-	-	4.4	6.7	-	∼150	J. Electrochem. Soc. 153 11 (2006) G956
		200		-	-	1.4	0.96			
		300		-	-	2.8	0.42			
TiN	NH_3_	320	4.9∼5	∼0	-	∼	1.0	-	142	J. Electrochem. Soc. 152 8 (2005) G589
TiN	N_2_/H_2_/Ar plasma	150	4.06	-	-	-	∼5.5	-	∼80	J. Korean Phys. Soc. 57 11 (2010) 806
		200	-	-	-	-	∼3			
		300	-	-	-	-	∼0.4			
		350	5.02				∼0.3			

**Figure 1:**
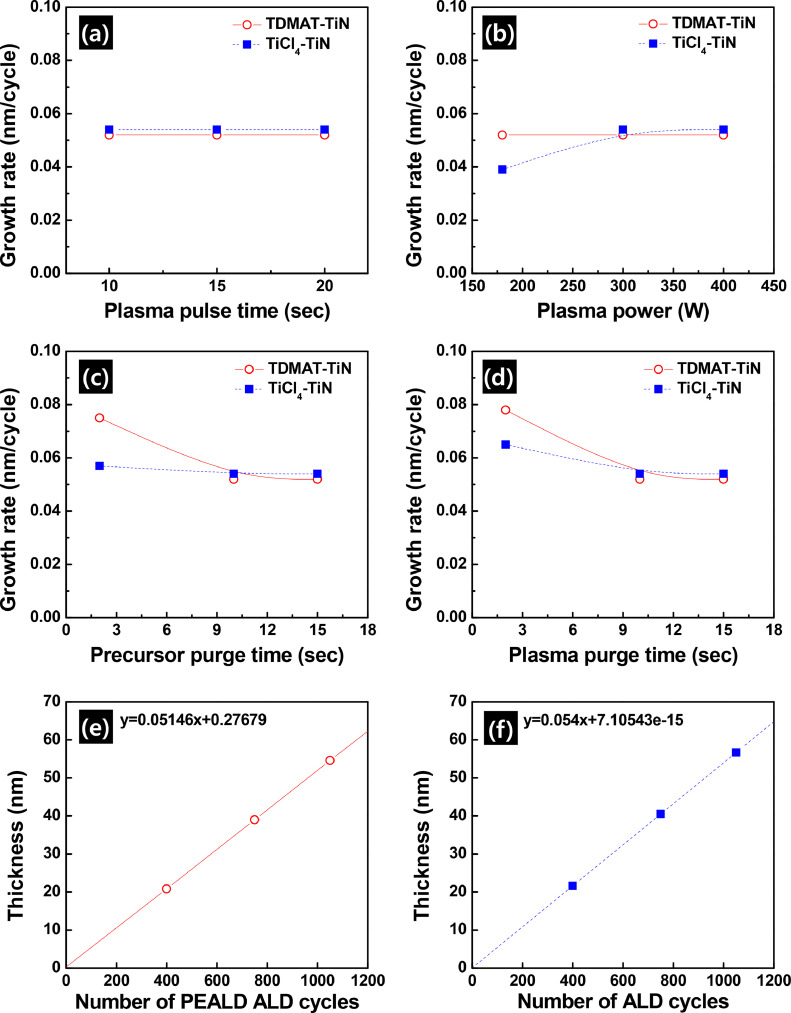
The growth rates of TDMT-TiN and TiCl_4_-TiN on SiO_2_ substrates, depending on (a) the plasma pulse time, (b) plasma power, (c) precursor purge time and (d) plasma purge time. The dependence of the film thickness (nm) on the number of PEALD cycles for (e) TDMAT-TiN and (f) TiCl_4_-TiN.

To confirm the self-limiting characteristics of TDMAT-TiN and TiCl_4_-TiN, the effects of growth parameters on the growth rates of TDMAT-TiN and TiCl_4_-TiN were systemically investigated. [2] Figure 1a shows the dependence of the growth rates of the films on the N_2_ plasma pulse time for TDMAT-TiN and on the N_2_/H_2_ mixed plasma pulse time for TiCl_4_-TiN at a fixed precursor pulse time of 1 sec, precursor and plasma purge times of 10 sec, and a plasma power of 300 W. For a given set of conditions, the growth rates of TDMAT-TiN and TiCl_4_-TiN were saturated at 0.052 nm/cycle and 0.054 nm/cycle, which confirms that 10 sec of plasma pulse time was sufficient to completely react with the adsorbed precursors. Figure 1b shows the growth rates of TDMAT-TiN and TiCl_4_-TiN thin films as a function of the plasma power. In these experiments, the precursor pulse time, precursor purge time, plasma pulse time, and plasma purge time were fixed at 1 sec, 10 sec, 10 sec, and 10 sec, respectively. In the case of TDMAT-TiN, a plasma power greater than 180 W was sufficient to complete the chemical reaction with the adsorbed precursors during the plasma pulse time of 10 sec, whereas a power significantly greater than 300 W was required for TiCl_4_-TiN for the same plasma pulse time. We subsequently investigated the effect of precursor and plasma purge times to clarify the complete removal of the volatile by-products after the precursor pulse and plasma pulse (Figure 1c, 1d). For a given precursor pulse time of 1 sec and a plasma pulse time of 10 sec with a plasma power of 300 W, the results related to the saturation behavior of the growth rates demonstrated that the volatile by-products were completely removed by 10 sec of a purge pulse. Thus, we assured that the self-limiting film growth for both TDMAT-TiN and TiCl_4_-TiN was achieved by adopting a sequential exposure of 1 sec of a precursor pulse, 10 sec of a purge pulse, 10 sec of a plasma pulse with a plasma power of 300 W, and 10 sec of a plasma pulse. Because of the self-limiting growth of TDMAT-TiN and TiCl_4_-TiN, the film thickness exhibits a linear dependence on the number of PEALD cycles, as shown in Figure 1e and 1f, and we can digitally control the desired film thickness with the number of PEALD cycles.

Table 2 shows structural information of two precursors. Projection area exhibits the lateral area occupied by one precursor molecule on the surface. Accordingly, distance of precursor perpendicular to the maximum projection can be calculated. According to the projection area and distance of precursor, TiCl_4_ molecular size is smaller than TDMAT.

**Table 2:** Molecular structures, calculated projection areas and distance perpendicular to the projection of the TiCl_4_ and TDMAT precursors.

Precursors	TiCl_4_	TDMAT
Structure (2D)		
Maximum projection area of molecules (Å^2^)	36.77	62.54
Distance perpendicular to the max projection (Å)	7.56	9.5

**Figure 2:**
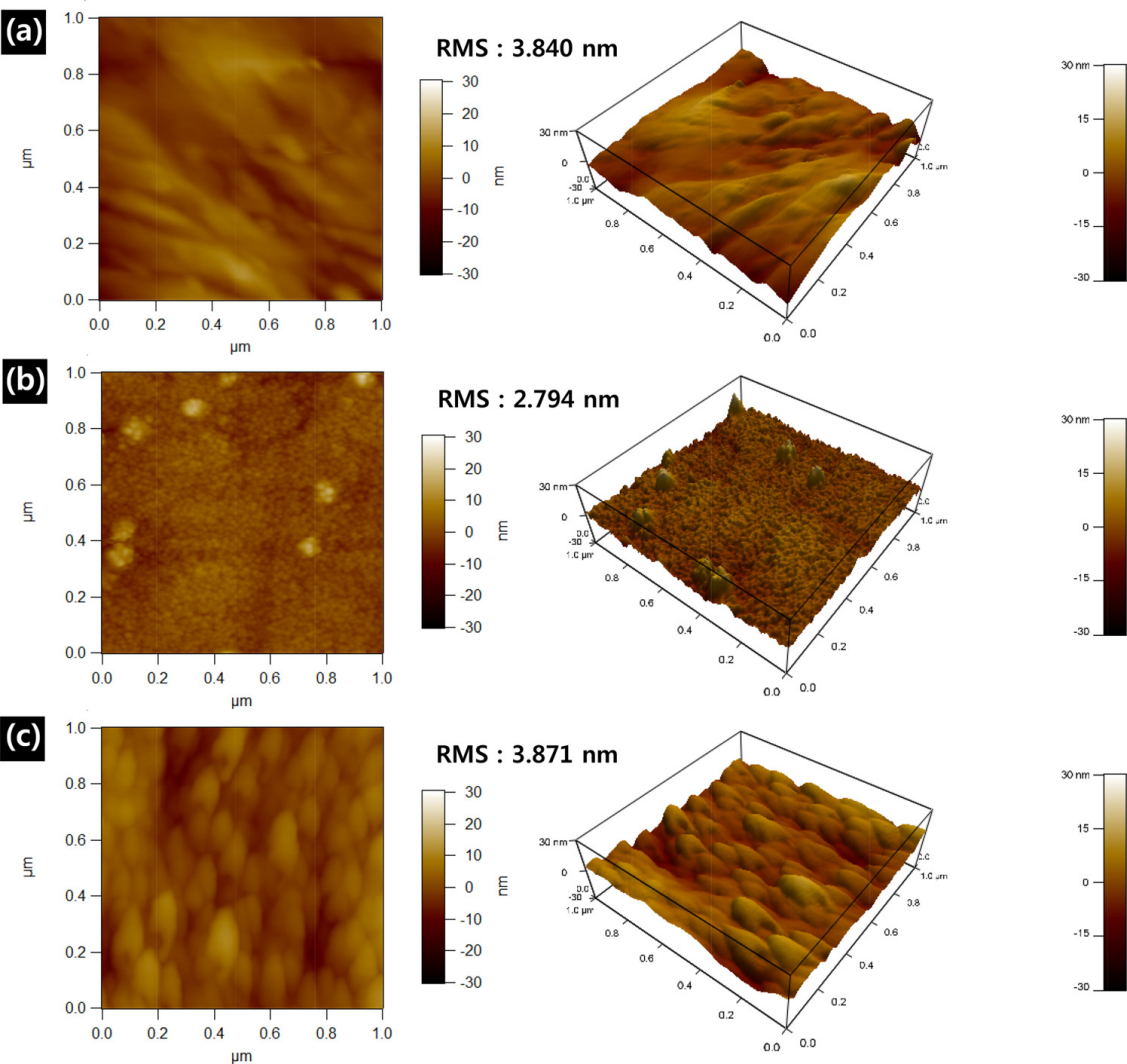
Atomic force microscopy images and corresponding 3D profiles of (a) the bare SS316L, (b) the TDMAT-TiN-coated SS316L and (c) the TiCl_4_-TiN-coated SS316L.

To quantitatively investigate the surface roughness before and after the PEALD-TiN coating of SS316L, we performed contact-mode atomic force microscopy to characterize the bare SS316L, TDMAT-TiN-coated SS316L, and TiCl_4_-TiN-coated SS316L with a scan area of 1 μm^2^, the results are shown in Figure 2. The TiN thin films were prepared with 1050 PEALD cycles using two precursors, TDMAT and TiCl_4_, on SS316L substrates. The measured root-mean-square (RMS) surface roughness values were 3.840 nm, 2.794 nm and 3.871 nm for the bare SS316L, TMDAT-TiN-coated SS316L and TiCl4-TiN-coated SS316L, respectively.

## 2 Experimental Design, Materials, and Methods

TiN thin films were prepared by the PEALD method using two types of precursors (i.e., TDMAT and TiCl_4_). To measure the overall film growth rate, we also used a 250-nm-thick SiO_2_/Si substrate whereas 0.2-mm-thick stainless steel 316L (SS316L) substrates was used for applying the bipolar plate. PEALD-TiN thin films were deposited at different growth temperatures of 200°C and 350°C for TDMAT and TiCl_4_, respectively. One deposition cycle of TiN using TDMAT consisted of a TDMAT precursor injection with 25 sccm Ar carrier gas, a purge pulse with 50 sccm Ar, a pulse for the N_2_ plasma exposure with 100 sccm N_2_ gas, and another 50 sccm Ar purge pulse. For the PEALD-TiN using TiCl_4_, the canister containing TiCl_4_ was maintained at a temperature of 25°C because of its high vapor pressure. Similarly, one deposition cycle of TiN using TiCl_4_ consisted of a TiCl_4_ precursor injection with 25 sccm Ar carrier gas, a purge pulse with 50 sccm Ar, a pulse for exposure to a mixed plasma with 100 sccm N_2_ and 20 sccm H_2_ gas, and another 50 sccm Ar purge pulse. During the PEALD-TiN processes using TDMAT and TiCl_4_, Ar gas was consistently supplied to the chamber at a flow rate of 50 sccm, and the chamber pressure was constantly maintained at 3 Torr. For the plasma pulse, radio-frequency (RF) plasma was used. More detailed experiment and condition are included in “Ultrathin Effective TiN Protective Films Prepared by Plasma-Enhanced Atomic Layer Deposition for High Performance Metallic Bipolar Plates of Polymer Electrolyte Membrane Fuel Cells”.

And, the film thickness was analyzed by field-emission scanning electron microscopy (FESEM, Hitachi, S-4800). Film morphology on SUS 316L was investigated by MFP-3D AFM (Asylum Research). Precursor structural information was simulated by Mavin program.

## Funding Information

This research was mainly supported by the Global Frontier R&D Program (2013M3A6B1078874) on Center for Hybrid Interface Materials (HIM) funded by the Ministry of Science, ICT & Future Planning, and by the National Research Foundation of Korea(NRF) grant funded by the Korea government(MSIT) (No. 2020R1A2C101484111).

## Declaration of Competing Interest

The authors declare that they have no known competing financial interests or personal relationships which have, or could be perceived to have, influenced the work reported in this article.

## Appendix Supplementary materials

Image, application 1

## References

[1] Lee W.-J., Yun E.-Y., Lee H.-B.-R., Hong S.W., Kwon S-H. (2020). Ultrathin Effective TiN Protective Films Prepared by Plasma-Enhanced Atomic Layer Deposition for High Performance Metallic Bipolar Plates of Polymer Electrolyte Membrane Fuel Cells. Appl. Surf. Sci..

[2] George S.M. (2010). Atomic Layer Deposition: An Overview. Chem. Rev..

