# Supplementary figures and images for: Dataset for TiN Thin Films Prepared by Plasma-Enhanced Atomic Layer Deposition Using Tetrakis(dimethylamino)titanium (TDMAT) and Titanium Tetrachloride (TiCl4) Precursor
Source: Data Brief. 2020 May 28;31:105777. doi: 10.1016/j.dib.2020.105777 (PMC7287241; doi:10.1016/j.dib.2020.105777)

## Slide 1
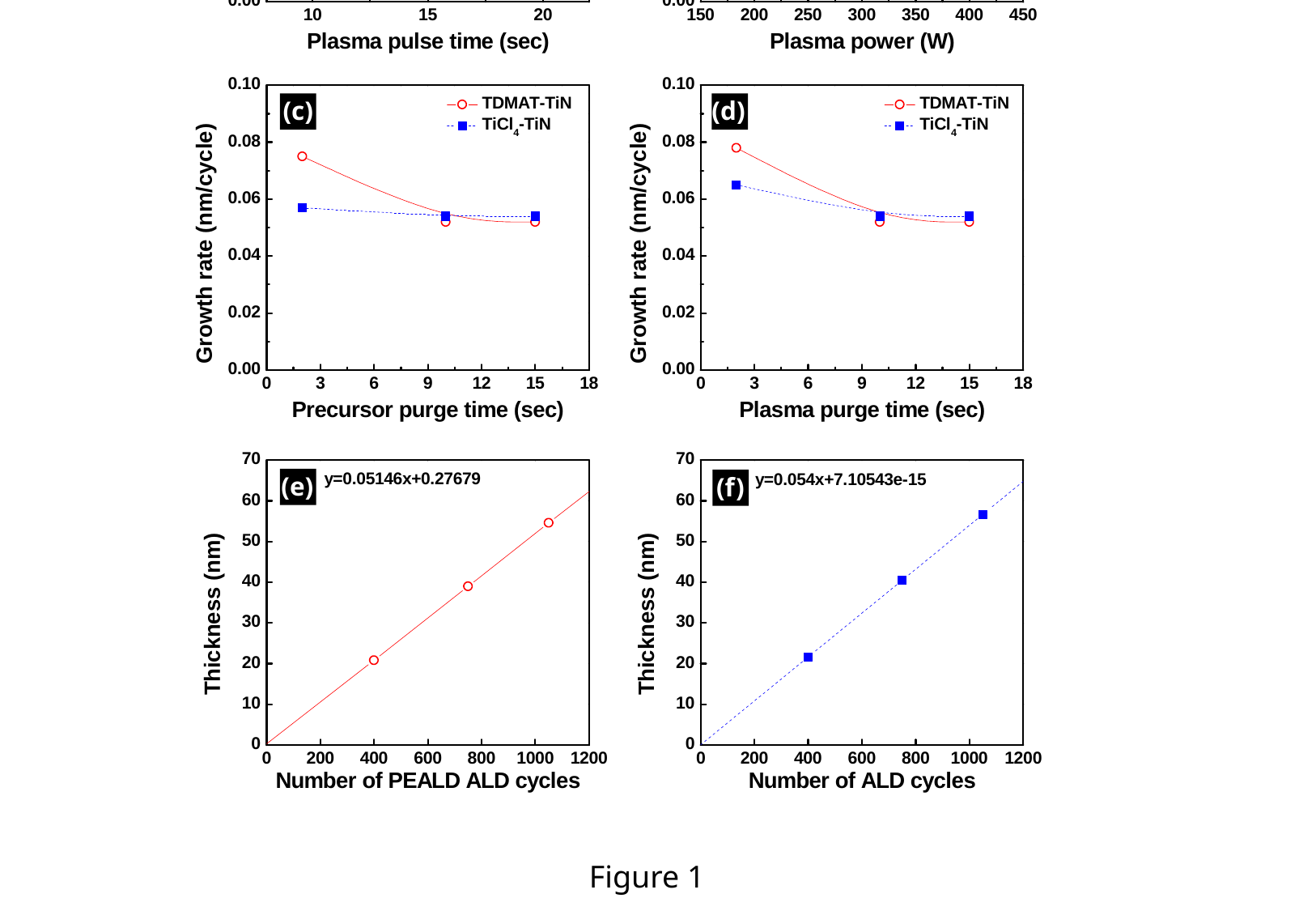

(a)
(b)
(c)
(d)
(e)
(f)
Figure 1

## Slide 2
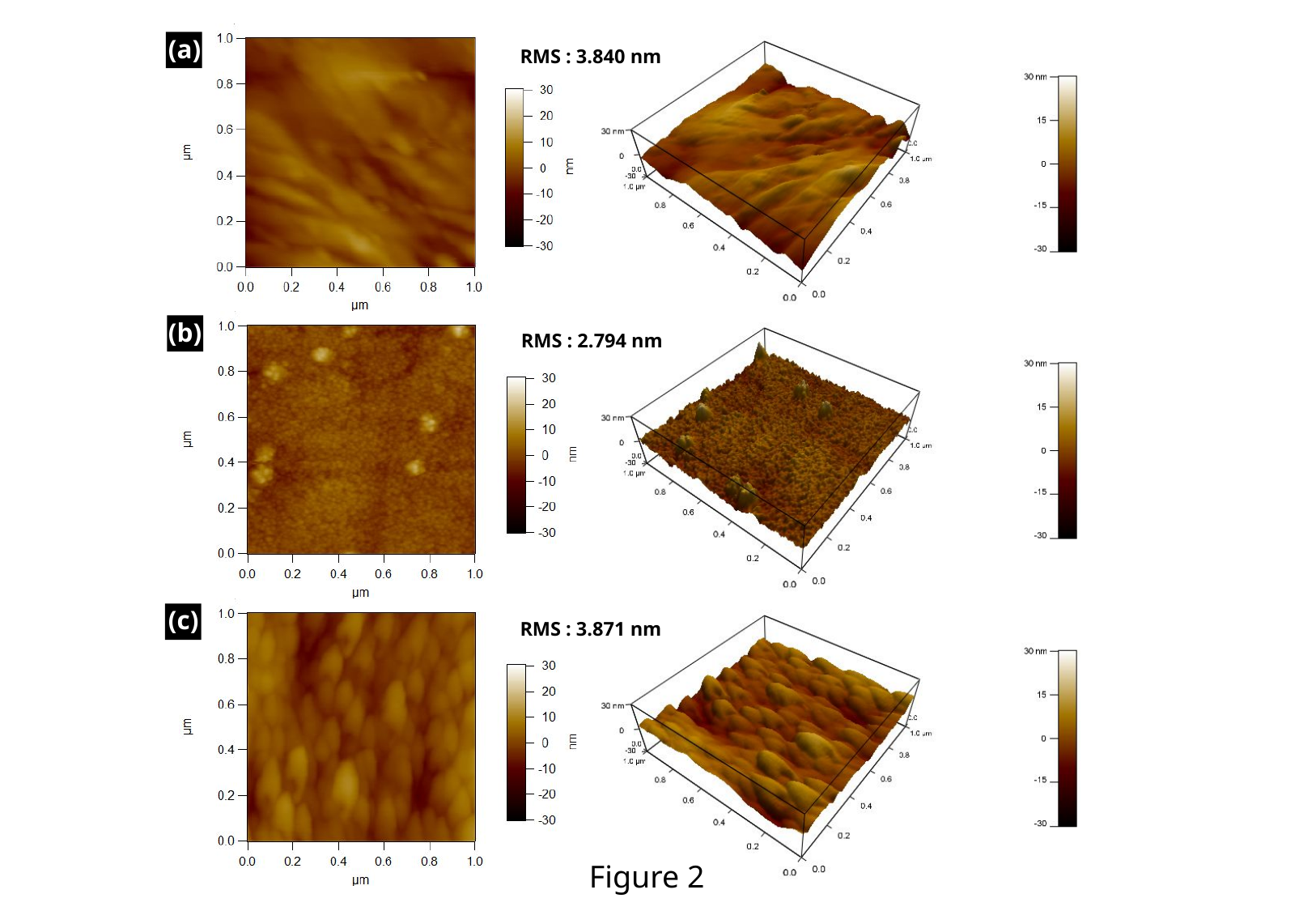

(a)
RMS : 3.840 nm
(b)
RMS : 2.794 nm
(c)
RMS : 3.871 nm
Figure 2

Supplement: Supplementary file 1 [file mmc1.pptx]
